# Anticipated synchronization in neuronal motifs

**DOI:** 10.1186/1471-2202-14-S1-P275

**Published:** 2013-07-08

**Authors:** Fernanda S Matias, Leonardo L Gollo, Pedro V Carelli, Mauro Copelli, Claudio R Mirasso

**Affiliations:** 1 Departmento de Física, Universidade Federal de Pernambuco, Recife, Pernambuco 50670-901, Brazil; 2 Instituto de Física Interdisciplinar y Sistemas Complejos, CSIC-UIB, Campus Univesitat de les Illes Balears E-07122 Palma de Mallorca, Spain; 3 Systems Neuroscience Group, Queensland Institute of Medical Research, Brisbane, QLD 4006, Australia

## 

Anticipated synchronization (AS) is a counterintuitive stable dynamical regime discovered by Voss in the last decade [1]. It consists in the stable synchronized regime between two identical autonomous dynamical systems coupled (unidirectionally) in a master-slave (MS) configuration, if the slave also receives a negative delayed self-feedback. In such regime the slave predicts the master. Although AS has shown to be stable in a variety of theoretical studies [1-3] and in experimental observations [4] with semiconductor lasers and electronic circuits, studies in biological and neuronal systems are still lacking.

The first verification of AS in neuronal models was done between two FitzHugh-Nagumo neurons coupled in a MS configuration with negative delayed self-feedback and driven by white noise [2]. In neuronal networks, AS means that the slave (post-synaptic) neuron can fire spikes right before the master (pre-synaptic) neuron. However the slave delayed self-feedback that induces the anticipated synchronization as suggested by Voss is unrealistic in neuronal circuitry.

Recently, it has been shown that in a biologically plausible neuronal model, the self-feedback can be replaced by an inhibitory loop mediated by an interneuron and chemical synapses [5]. This master-slave-interneuron motif exhibits the usual delay synchronized (DS) regime for small values of the inhibitory synaptic conductance (*g*) as well as the AS regime for larger *g*. Moreover, the time delay between consecutive spikes of the master and the slave neurons is a function of the synaptic conductances. Both regimes were shown to be stable for a large set of parameters, different external currents, structural motif and neuron models [6]. Here we investigate the robustness of AS for others motifs, larger populations, neuronal heterogeneity and noise. In all the situations the transition from DS to AS is continuous and smooth.
